# 3D Matrices for Enhanced Encapsulation and Controlled Release of Anti-Inflammatory Bioactive Compounds in Wound Healing

**DOI:** 10.3390/ijms24044213

**Published:** 2023-02-20

**Authors:** Raluca Nicu, Diana Elena Ciolacu, Anca-Roxana Petrovici, Daniela Rusu, Mihaela Avadanei, Andreea Cristina Mihaila, Elena Butoi, Florin Ciolacu

**Affiliations:** 1 Department of Natural Polymers, Bioactive and Biocompatible Materials, “Petru Poni” Institute of Macromolecular Chemistry, 700487 Iasi, Romania; 2 Center of Advanced Research in Bionanoconjugates and Biopolymers, “Petru Poni” Institute of Macromolecular Chemistry, 700487 Iasi, Romania; 3 Department of Physics of Polymers and Polymeric Materials, “Petru Poni” Institute of Macromolecular Chemistry, 700487 Iasi, Romania; 4 Department of Physical Chemistry of Polymers, “Petru Poni” Institute of Macromolecular Chemistry, 700487 Iasi, Romania; 5 Biopathology and Therapy of Inflammation, Institute of Cellular Biology and Pathology “Nicolae Simionescu”, 050568 Bucuresti, Romania; 6 Department of Natural and Synthetic Polymers, “Gheorghe Asachi” Technical University of Iasi, 700050 Iasi, Romania

**Keywords:** cellulose, dextran, polyphenols, hydrogels, controlled release, wound healing

## Abstract

Current trends in the development of wound dressings are oriented towards the use of biopolymer-based materials, due to their unique properties such as non-toxicity, hydrophilicity, biocompatibility and biodegradability, properties that have advantageous therapeutic characteristics. In this regard, the present study aims to develop hydrogels based on cellulose and dextran (CD) and to reveal their anti-inflammatory performance. This purpose is achieved by incorporating plant bioactive polyphenols (PFs) in CD hydrogels. The assessments include establishing the structural characteristics using attenuated total reflection Fourier transformed infrared (ATR-FTIR) spectroscopy, the morphology by scanning electron microscopy (SEM), the swelling degree of hydrogels, the PFs incorporation/release kinetics and the hydrogels’ cytotoxicity, together with evaluation of the anti-inflammatory properties of PFs-loaded hydrogels. The results show that the presence of dextran has a positive impact on the hydrogel’s structure by decreasing the pore size at the same time as increasing the uniformity and interconnectivity of the pores. In addition, there is an increased degree of swelling and of the encapsulation capacity of PFs, with the increase of the dextran content in hydrogels. The kinetics of PFs released by hydrogels was studied according to the Korsmeyer–Peppas model, and it was observed that the transport mechanisms depend on hydrogels’ composition and morphology. Furthermore, CD hydrogels have been shown to promote cell proliferation without cytotoxicity, by successfully culturing fibroblasts and endothelial cells on CD hydrogels (over 80% viability). The anti-inflammatory tests performed in the presence of lipopolysaccharides demonstrate the anti-inflammatory properties of the PFs-loaded hydrogels. All these results provide conclusive evidence on the acceleration of wound healing by inhibiting the inflammation process and support the use of these hydrogels encapsulated with PFs in wound healing applications.

## 1. Introduction

Wound healing consists of four partially overlapping stages, namely hemostasis, inflammation, proliferation, and remodeling, which determine whether the wound will heal normally or will undergo an aberrant healing process that will finally lead to fibrosis [1]. Thus, the inflammation is one of the key stages of the wound healing process, extending over a period of 3 to 7 days. Its rigorous control is crucial so that this process does not become pathogenic, with bacterial colonization and excessive formation of pathological scar tissue [2].

For this reason, the choice of an appropriate dressing for wounds is of crucial importance, and it must present some special characteristics, such as: (i) an adequate water retention, to be able to absorb exudates; (ii) adequate mechanical stability to support skin lesions; (iii) resistance to breaking; (iv) antibacterial properties, to prevent biofilm formation, and (v) promoting healing, by releasing a long-term drug and growth factors [3]. Thus, the role of wound dressings is to cover, clean and protect the wound from the external environment.

Among the wound dressings, hydrogel dressings have become the main focus in wound healing research, because they can maintain a moist environment at the wound interface, allow gaseous exchange, act as a barrier against the invasion and proliferation of microorganisms, remove excess exudates, have excellent biocompatibility and be easily removed without trauma [4,5]. By incorporating different anti-inflammatory agents, they can also demonstrate anti-inflammatory capabilities [6].

In recent decades, research has focused on wound-healing applications of hydrogels based on natural polymers, because these are abundantly available and inexpensive, and are considered to have great potential for large-scale production. However, in many cases, the use of a single type of natural polymer cannot meet the various requirements of an ideal wound dressing, and therefore binary, ternary or multicomponent polymeric mixtures, that have synergistic interactions, are generally used [7,8,9,10].

**Figure 1:**
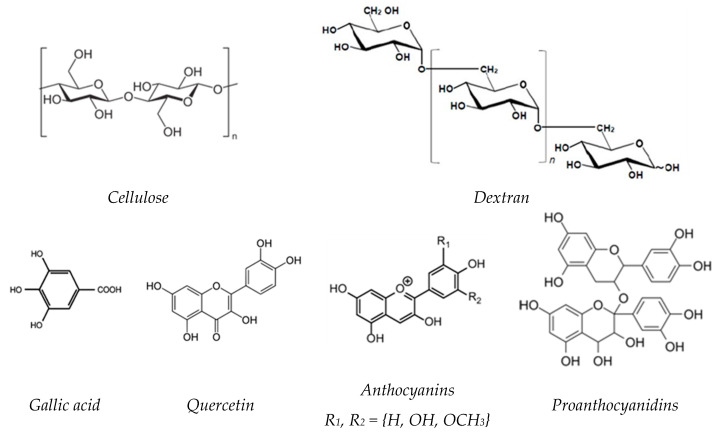
Chemical structure of cellulose, dextran and the major phenolic compounds identified in grape seeds.

In the present study, an attempt was made to develop a binary mixture of polysaccharides, using cellulose and dextran. These polysaccharides are both composed of D-glucose units, but differs in the way the units are arranged and connected by linear and side-chain links (Figure 1) [11,12].

Cellulose-based hydrogels are especially distinguished by their excellent mechanical properties and thermal stability, while dextran-based hydrogels are highly hydrophilic and have a great ability to support cell adhesion and proliferation, but exhibit poor mechanical properties [13,14,15,16,17,18]. Thus, the combination of the unique properties of these two polysaccharide-based hydrogels can result in obtaining a perfect wound dressing material.

In the literature, there are only a few studies related to the hydrogels based on cellulose and dextran, and these refer either to (i) dextran and bacterial cellulose (BC) [5], or to (ii) dextran sulfate (DS) and carboxymethyl cellulose (CMC) [19], (iii) amino dextran and CMC [20] and (iv) cellulose nanocrystals (CNC), oxidized dextran and gelatin [21]. The performance in wound healing applications of the dextran/BC hydrogel reveals that the addition of dextran affected the network structure of BC, resulting in the decrease of the water content; cell-based experiments showed that these hydrogels promoted enhanced cell proliferation, without cytotoxicity compared to unmodified BC, while in vivo wound healing tests demonstrated that the hydrogels can accelerate the wound healing process [5]. Hydrogel composite beads based on CMC and DS were prepared using ionotropic gelation, in presence of sodium n-dodecyl sulfate. By increasing the DS content, a remarkably improvement of the adsorption performances was observed, as well as a good reusability after five adsorption/desorption cycles [19]. Another example of composite hydrogel is that obtained by conjugation of CMC with amino dextran (DEX), in order to formulate piroxicam loaded CMC-DEX hydrogels for topical application. The entrapment efficiency was observed as being 87.36 ± 1.23% for CMC-DEX and 72.35 ± 2.35% in the case of CMC hydrogels, respectively. The piroxicam deposited from CMC hydrogels was 15.61 ± 1.5 mg/mL (epidermis) and 6.24 ± 1.2% (dermis) in 12 h, while in the case of CMC-DEX hydrogels, the piroxicam retention was 5.21 ± 1.19 mg/mL (epidermis) and 26.34 ± 0.5 mg/mL (dermis), in 12 h. This fact proved that when there was a cross-linking reaction between DEX and CMC, it increased the penetration of piroxicam [20]. The hydrogels obtained from cellulose nanocrystals (CNC), oxidized dextran and gelatin were fabricated by 3D printing [21].

No study has reported the preparation of chemically cross-linked hydrogels based on cellulose and dextran by a two-step method, dissolution in alkaline solutions followed by cross-linking in the presence of epichlorohydrin.

Numerous pharmacological agents can significantly reduce or completely inhibit the inflammation process, thus promoting wound healing [22,23].

In the last few years, there has been a growing interest in compounds from natural sources with anti-inflammatory properties, among them polyphenols (PFs) which have attracted significant attention. PFs are a large group of organic compounds distributed in various plants and marine organisms, covering a wide range of complex structures (Figure 1) [24,25]. They are classified according to their chemical structures into flavonoids such as flavones, flavonols, isoflavones, neoflavonoids, chalcones, anthocyanidins, and proanthocyanidins and non-flavonoids, such as phenolic acids, stilbenoids and phenolic amides [26].

PFs have been the subject of numerous studies, being tested for their unique antioxidant [27,28] and anti-inflammatory properties [26,29,30]. The ability of these PFs to modify the expression of several pro-inflammatory genes including those encoding cytokines, lipoxygenase, nitric oxide synthases cyclooxygenase, in addition to their antioxidant characteristics, such as reactive oxygen species scavenging (ROS), contributes to the regulation of inflammatory signaling [26]. Moreover, the incorporation of these polyphenolic compounds as therapeutic agents in wound dressings makes it possible to repair wounds faster and better [27,31].

In this regard, the present study aims to prepare new 3D matrices based on cellulose and dextran with anti-inflammatory activity, capable of encapsulation/controlled release of PFs, considering the applications in wound healing. Lipopolysaccharides (LPS), the major component of Gram-negative bacteria cell walls that can cause an acute inflammatory response, were used as a pro-inflammatory model to reveal the protective mechanism of PFs in LPS-induced inflammation.

## 2. Results and Discussion

### 2.1. Swelling Behavior of Hydrogels

One of the most important characteristics of hydrogels is the degree of swelling because all the other properties of hydrogels are related to this value. The hydrogel’s polymeric network can absorb 10 to 1000 times its dry weight in water, due to the hydration of the hydrophilic groups and the penetration of the water molecules into the pores of the 3D matrices [32].

In our particular case, the equilibrium swelling degree (Q_eq_, %) of never-dried hydrogels varies from 1640% for cellulose hydrogels (C) up to 5310% for dextran hydrogel (D). Regarding the cellulose-dextran (CD) hydrogels it was observed that even by adding the lowest dextran content (CD75/25 hydrogel, namely, a dextran content of 25%) there was almost a doubling of the swelling degree compared to C hydrogel (more precisely, from 1640% to 2980%). The swelling degrees of CD hydrogels gradually increase with the increase of the dextran content in the 3D network, but not higher than that of the D hydrogel. The Q_eq_ data obtained for CD hydrogels demonstrate their superabsorbent character.

The composition and Q_eq_ of the hydrogels, as well as the gel fraction yield are presented in Table 1.

The maximum swelling degree (Q_max_, %) of the freeze-dried hydrogels increases from approximately 1060% for C hydrogel up to 3740% for D hydrogel (Table 2). For the other five CD hydrogel formulations, which contain different proportions of cellulose and dextran, Q_max_ is between 1485 and 2367%, with the observation that it increases with the increase of the dextran content in the hydrogels’ matrix.

**Figure 2:**
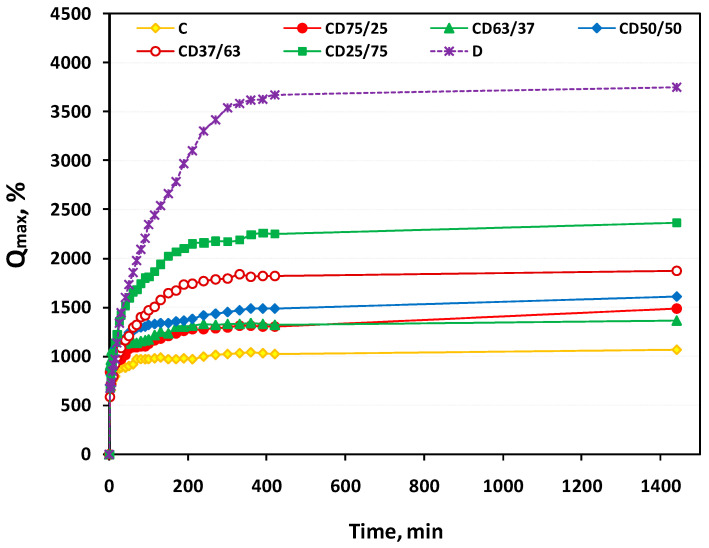
Swelling kinetics curves of the hydrogels, as a function of composition.

The evolution in time of the Q_max_ of the C, D and CD hydrogels, as a function of composition, is presented in Figure 2.

**Figure 3:**
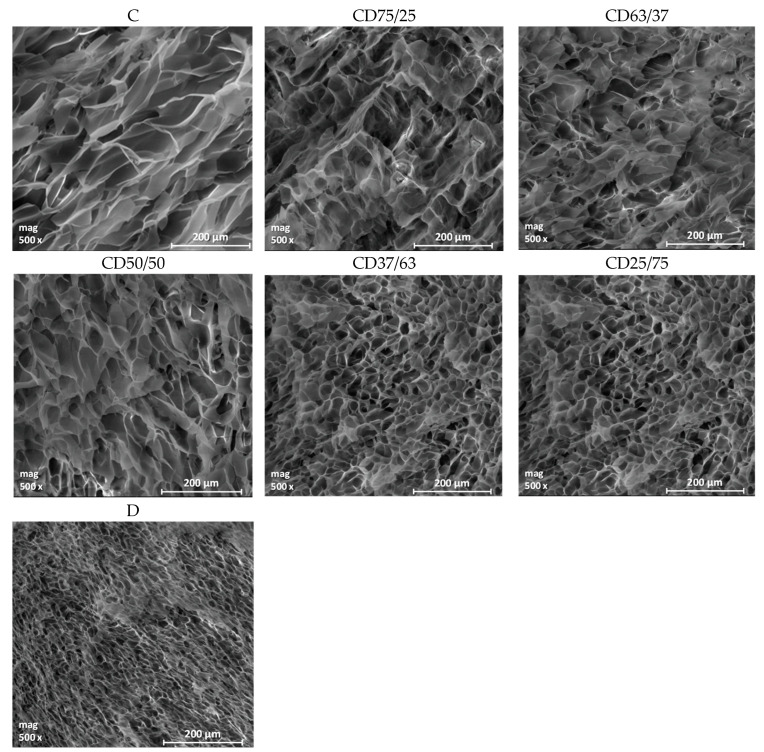
SEM images of the hydrogels obtained from cellulose (C), dextran (D) and their binary mixtures, in different gravimetric ratios (CD).

As a general observation for all hydrogels, there was a sharp increase of Q_max_ at the beginning of the swelling process, after which the increase continued until reaching a plateau. This behavior depends on the hydrogel’s composition, more exactly on the content of dextran in the 3D matrix. Thus, if the equilibrium appears quite quickly in the case of C hydrogel, approximately 20 min, the time to reach the Q_max_ increased with increasing of the content of dextran in the matrix. Unlike C hydrogel, the Q_max_ of D hydrogel is reached after approximately 300 min. This different swelling behavior is obviously due to the different morphology of these 3D matrices, a major influence in this regard being the dimensions of the pores and their interconnectivity in the porous network of the hydrogels (see Figure 3).

In order to study the effect of the hydrogel composition on the kinetics of the water uptake process, the empirical Equation (3) [33] was used and the swelling kinetic parameters of C, D and CD hydrogels are presented in Table 2.

The swelling process of the hydrogels is a complex process involving three successive steps: (i) the water molecules diffuse into the polymer network, followed by (ii) the relaxation of hydrated polymer chains, and then, in the final stage, (iii) the expansion of the polymer network into the surrounding aqueous solution [34]. Depending on the value of the diffusional exponent (*n*) and the geometry of the hydrogels (i.e., thin films, cylinders or spheres), the water transport mechanism in the hydrogel network can be established [35].

In our particular case of cylinder hydrogels, the values of the diffusional exponent (*n_sw_*) are lower than 0.45, indicating the fact that the water transport mechanism follows the pseudo (less) Fickian diffusion, characterized by a diffusion rate lower than the relaxation rate of the polymer network. However, with the increase of the dextran content in the hydrogel formulations, a slight increase of the *n_sw_* value is observed, showing a tendency towards the perfectly Fickian diffusion mechanism (*n_sw_* = 0.45), where the polymer chains have a higher mobility that allows easier penetration of the solvent [34].

The swelling rate constant, *k_sw_*, is an important parameter that characterizes the diffusion rate in a hydrogel network. It can be seen that this constant varies with the hydrogel’s composition; more precisely, this parameter slightly increases with the increase of dextran content in hydrogel matrices.

For all hydrogels, the correlation coefficients R^2^ are higher than 0.99, indicating a good fitting between experimental data and the chosen model.

### 2.2. Hydrogels Morphology

The cross-sectional morphologies of the cellulose-dextran hydrogels were analyzed by using scanning electron microscopy (SEM), which offers information regarding the pores’ appearance and the structural uniformity of the hydrogels.

As expected, considering the various swelling degrees of the hydrogel formulations, the analysis of SEM micrographs evidenced different morphologies, depending on the matrix composition (Figure 3). Thus, if the cellulose-based hydrogel (C) has a discontinuous structure with large, irregular pores, in the case of the dextran-based hydrogel (D) a homogeneous structure with small, interconnected pores is observed. Related to CD hydrogels, it was observed that by increasing the dextran content in the hydrogels’ 3D network, the size and unevenness of the pores decreases. The average pore size decreases from 66 μm for C hydrogels, to 41.6 μm for CD50/50 hydrogel and to 13.5 μm for D hydrogel (Table 3).

Regarding the pore size homogeneity, this increases with increasing dextran content in the hydrogels, a fact which is also reflected by the standard deviation (SD). Thus, the less homogenous structure was recorded for C hydrogel, with the highest SD value (±12) and by increasing the dextran content, this value gradually decreased to that for the D hydrogel (±3), which has the most uniform structure. In addition, although the network of the D hydrogel has small pores, compared to those of C hydrogel, its pores are uniformly distributed within the 3D network of hydrogels and are interconnected. This fact is shown by the high swelling degree of this hydrogel, which further influences its capacity for encapsulation and release of bioactive compounds, respectively. The only disadvantage of D hydrogel is the low gel fraction yield, but this can be controlled by its combination with cellulose, thus obtaining materials with optimal properties in wound healing.

### 2.3. ATR-FTIR Measurements

The ATR-FTIR spectroscopy technique was used to analyze the structural changes of the CD hydrogels, obtained after chemical cross-linking the two polysaccharides in different proportions, but also to confirm the incorporation of polyphenolic compounds in the hydrogel matrix.

#### 2.3.1. ATR-FTIR Measurements for CD Hydrogels

**Figure 4:**
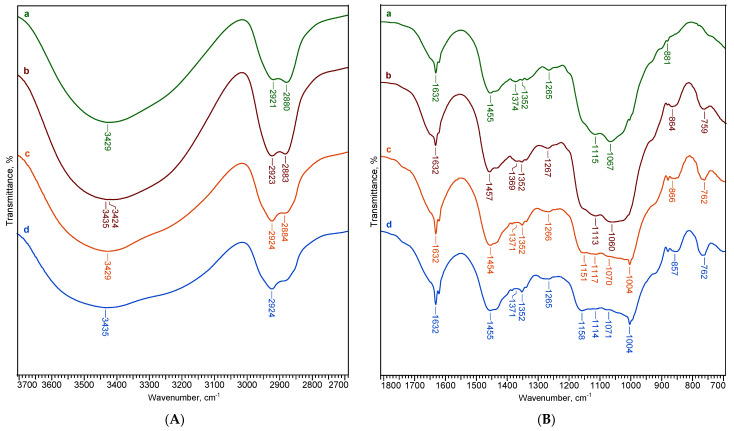
ATR-FTIR spectra in the regions of (**A**) 3700–2700 cm^−1^ and (**B**) 1800–700 cm^−1^, for the hydrogels based on cellulose (a), dextran (d), and cellulose-dextran hydrogels: CD50/50 (b) and CD25/75 (c).

The ATR-FTIR spectra of cellulose and dextran hydrogels, as well as the spectra of two of the bicomponent CD hydrogels (CD50/50 and CD25/75, respectively) are presented in Figure 4 (to simplify the figure, we chose only two bicomponent hydrogels). The two polysaccharides, cellulose and dextran, show quite similar spectra, but each one is still distinguished by their characteristic absorption bands. Generally, the absorption bands for polysaccharides are grouped in two main wavenumber regions, namely the region of 3700–2700 cm^−1^ and 1800–400 cm^−1^, respectively.

In the range of 3660–2700 cm^−1^ are presented the broad band at 3429–3435 cm^−1^ characteristic for O-H stretching vibrations of the OH groups and the band at 2921 cm^−1^, attributed to skeletal C-H stretching vibration in polysaccharides [36]. The bands located at 1631 cm^−1^ correspond to the O-H bending of adsorbed water.

In the ATR-FTIR spectrum of cellulose, there is a particular region between 1550 cm^−1^ and 800 cm^−1^ called “fingerprint” region, where the main absorption bands are: 1455 cm^−1^ assigned to CH_2_ bending vibration, 1374 cm^−1^ and 1352 cm^−1^ to C-H bending and in-plane OH bending, 1265 cm^−1^ to C-O-H in plane bending, 1114 cm^−1^ to C–O–C anhydroglucose ring asymmetric stretching, 1066 cm^−1^ to C-O stretching and the band at 881 cm^−1^ assigned to C-O-C stretching at the β-(1–4)-glycosidic linkage [37,38,39].

The main absorption bands that characterize the dextran were found at 1158 cm^−1^, being related to the vibrations of the glycosidic linkage C-O-C of α-d-glucopyranoses, while the band at 1003 cm^−1^ is assigned to the vibration of the C-O bond of α-d-glucoses and chain flexibility around the α-(1→6) glycosidic bond [40,41]. The shoulder around 912 cm^−1^ along with the band at 857 cm^−1^ indicate the existence of glycosidic links, a branched chain in α-(1→3) anomeric configuration [12,42]. Moreover, in the case of dextran, the band at 761 cm^−1^ is considered a reference band, against which the structural changes that take place following the reaction can be determined [43].

In order to quantitatively characterize the changes produced in the cellulose matrix after mixing with dextran in different proportions, some absorption ratios of the characteristic bands were used and listed in Table 4. For instance, the hydrogen bond intensity (HBI) is correlated with the high ordered phase (crystalline) and degree of intermolecular regularity [36,44]. This parameter was determined from the ratio A_3429_/A_1336_, where the band at 3429 cm^−1^ is assigned to O–H stretching vibrations, while the band at 1336 cm^−1^ is due to C–O-H in-plane bending vibration [33,39].

Increasing the dextran content in the cellulosic matrix led to a decrease in the HBI values, indicating a decrease in the samples’ crystallinity. This fact is also confirmed by a gradual decrease of the maximum absorbance of OH stretching from the spectra of cellulose hydrogel to that of dextran hydrogel and implicitly of the total area of these peaks (Figure 4A).

The proof of the chemical cross-linking reaction in hydrogels is illustrated in the FTIR spectra by the presence of absorption bands at 1116 cm^−1^ (deformation vibration of ether linkages) and 1066 cm^−1^ (primary and aliphatic ethers groups), corresponding to the stretching vibrations of the C-O and C-O-C ether bonds [33]. In order to highlight the crosslinking capacity of the cellulose (FTIR internal standard: 2900 cm^−1^) and dextran (FTIR internal standard: 761 cm^−1^), the following ratios were established: A_1116_/A_2900_, A_1066_/A_2900_, A_1116_/A_761_ and A_1066_/A_761_. The hydrogels’ crosslinking capacity obviously decreases with the increase of the dextran content in the hydrogel’s matrix, confirming the increased ability of cellulose to crosslink in the presence of EPC, compared to dextran. The obtaining of a more relaxed 3D network of the hydrogels with increasing dextran content was also confirmed by the data obtained from the hydrogels’ gel fraction yield (from 99.9% for cellulose to 76.3% for dextran) and the swelling data (Table 1 and Table 2).

Table 4 also includes the A_1630_/A_2900_ ratios, associated with the ability of polysaccharides to bond water molecules into their network. These ratios increase with the increasing of dextran content in the hydrogel matrix, reflecting the higher hydrophilic character of dextran hydrogels compared to cellulose ones. This observation is also confirmed by the data on the swelling degrees of the hydrogels.

#### 2.3.2. ATR-FTIR Measurements for PFs-Loaded Hydrogels

**Figure 5:**
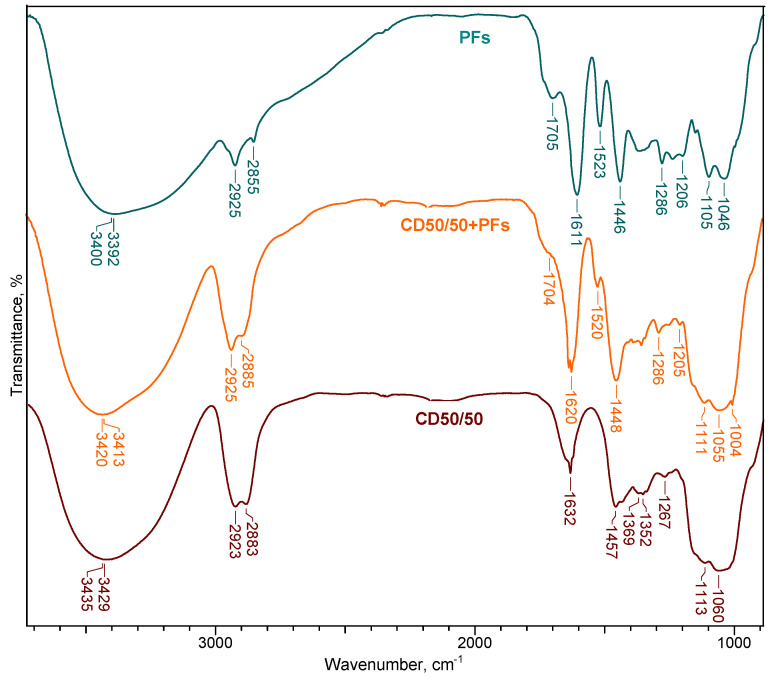
ATR-FTIR spectra of PFs, CD50/50 hydrogel and PFs-loaded hydrogel: CD50/50 + PFs.

Figure 5 shows the ATR-FTIR spectra of CD50/50 hydrogel loaded with PFs (CD50/50 + PFs) compared with the spectrum of PFs alone and CD50/50 hydrogel without PFs, respectively.

The main characteristic ATR-FTIR absorption bands of PFs are: 3391 cm^−1^ for OH stretching vibration, 2924 cm^−1^ for CH stretching vibration, 1705 cm^−1^ for C-O stretching vibration, 1611 cm^−1^ for C-C stretching vibration, 1445 cm^−1^ for CH_2_ bending vibrations, 1285 cm^−1^ and 1206 cm^−1^ for C-O stretching vibration (aryl ether band), 1105 cm^−1^ for C-O-C stretching vibration (alkyl ether band) and 1046 cm^−1^ for C-O stretching vibration (alkyl substituted ether) [33].

It is observed that, in addition to the characteristic absorption bands of CD50/50 hydrogel, the CD50/50 + PFs spectra contain also the specific bands of polyphenolic compound, proving their successful incorporation in the hydrogel’s network.

For instance, the absorption band at 1705 cm^−1^ in PFs, assigned to C=O stretching vibrations of carbonyl double bond, is shifted to 1704 cm^−1^ for the PFs-loaded hydrogel. The band at 1522 cm^−1^, corresponding to in-plane CH bending vibration from the phenyl rings, is shifted to lower wavenumbers, 1519 cm^−1^, after PFs incorporation in CD50/50 hydrogel. The absorption bands at 1285 cm^−1^ and 1206 cm^−1^ from the FTIR spectrum of PFs are also found in the spectrum of CD50/50 + PFs, but with lower intensities. Regarding the bands 1105 cm^−1^ and 1046 cm^−1^ from PFs spectrum, they are also found in the CD50/50 + PFs spectrum, but are shifted to higher wavenumbers (1110 cm^−1^ and 1055 cm^−1^) and overlapped by the characteristic bands of CD hydrogel (1112 cm^−1^ and 1059 cm^−1^).

Two ratios of the characteristic absorption bands intensity of PFs, namely A_1522_/A_2900_ and A_1705_/A_2900_, were used to quantitatively evaluate the incorporation of PFs in the hydrogels’ matrix (Table 5). Both ratios increase with increase in the PFs content in the hydrogels and the obtained data are in good correlation with the incorporation degree (I_d_).

### 2.4. In Vitro Release of the Polyphenolic Compound from Hydrogels

**Figure 6:**
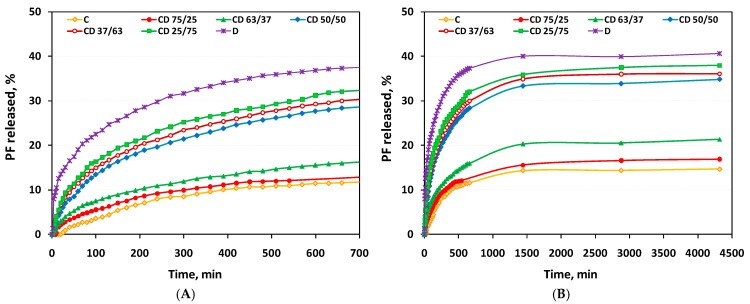
The release profiles of PFs from C, D and CD hydrogels, for (**A**) 12 h and (**B**) 72 h.

The release profiles of PFs indicate a prolonged release of them from CD hydrogels (over a period of 3 days), a release that depends on the composition of the hydrogels (Figure 6). As can be seen, all CD hydrogels show a sustained PFs release which constantly increases until about 1400 min (24 h), after which it stabilizes on a plateau. The PFs release is presented for two different time periods, (i) the first 12 h, where the clear influence of the hydrogels’ composition on the PFs release rate can be observed (Figure 6A), and (ii) the release up to 72 h, in order to highlight the plateau phase (Figure 6B). The initial release can be explained as the release of PFs trapped on the surface and in the pores in its immediate vicinity, during the incorporation process.

The PFs release is higher with increased dextran content in the matrix. For instance, for the first 30 min, the PFs release was only 6.3% (relative to the total PFs released) for cellulose hydrogels, while for CD50/50 hydrogels the release was 20.2% and for CD25/75 it was 24.7%, respectively. As expected, a higher swelling degree of dextran hydrogel (D) leads to more uptake of the drug during loading and furthermore, to a higher PFs release after only 30 min, of 37.2%.

Related to the time necessary to reach the maximum percentage of PFs released (72 h), the highest amount of PFs release is registered for the D hydrogel, reaching up to 40.6% (relative to the total incorporated amount of PFs), while for C hydrogel, this is only 14.7%. The sustained release of PFs continues even after 72 h, up to 14 days (results not shown), reaching for D hydrogels up to 45.01% and for C hydrogels up to 20.9%, respectively.

To understand the release behavior for PFs and to evaluate the possible release mechanism of PFs from the hydrogels, the experimental release data were investigated by fitting the Korsmeyer–Peppas model using the empirical Equation (5) [35]. This equation was used for the linearization of the release data and *n* and *k* values were calculated from the slope and intercept of the plot of ln(M_t_/M_∞_) against ln(t) [45]. Their values are presented in Table 6, along with the regression coefficients, R^2^. The diffusional exponent, *n*, is used as an indicator for the release mechanism and the kinetic constant, *k*, is used to describe the structural and geometrical parameters of the hydrogels. For cylindrical hydrogels, if the exponent *n* < 0.45, the drug release mechanism is a Fickian diffusion, while if 0.45 < *n* < 0.89, then it is a non-Fickian or anomalous diffusion; when *n* > 0.89, the release is a Case II transport mechanism [35].

By using this model, the regression coefficient was found very close to unity (R^2^ = 0.984−0.994), suggesting that the release data best fit to the Korsmeyer–Peppas model. The *n* value of the Korsmeyer–Peppas model was further used to examine the PFs release mechanism. Thus, the *n* values range from 0.329 for D hydrogel to 1.004 for C hydrogel, while for CD hydrogels *n* falls between these two values, more exactly to 0.504 < *n* < 0.573, depending on the hydrogel’s composition. The different values obtained for the diffusional exponent, *n,* indicate different diffusion mechanisms for PFs-loaded hydrogels. For instance, for PFs-loaded D hydrogel, the *n* < 0.45 indicates a pseudo (less) Fickian mechanism that is diffusion-controlled and is characterized by a high diffusion rate and a low rate of polymer chain relaxation [46]. Instead, the *n* value obtained for C hydrogel (*n* = 1.004) indicates a non-Fickian mechanism (Case II) that is controlled by polymer relaxation and the release rate is constant. The PFs loaded CD hydrogels (0.45 < *n* < 0.89) exhibit an anomalous (non-Fickian) transport mechanism, when the solvent diffusion rate and the rate of polymer chain relaxation are comparable [35].

Based on these results, it can be concluded that PFs release is usually controlled by a diffusion process and so, the release profile can be easily modified by changing the material properties, including the pore size or pore connectivity, which are very closely correlated with the hydrogels composition.

### 2.5. Cytocompatibility and Anti-Inflammatory Tests

#### 2.5.1. Cytocompatibility of CD Hydrogels with Human Cells

**Figure 7:**
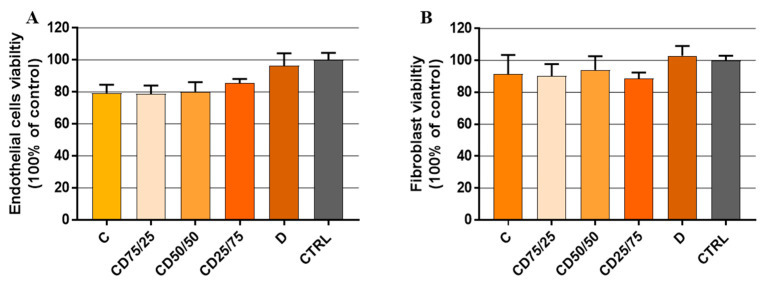
The viability of endothelial cells (**A**) and fibroblasts (**B**), after cultivation on different CD hydrogels, was determined by XTT assay. Cells were seeded for 72 h on the hydrogels or in a normal TPC culture plate (CTRL). Results are expressed as a mean ± standard deviation (SD) (*n* = 3).

The safety of the wound dressing is the top priority for its clinical efficiency [5]. Recent data showed that endothelial cells and fibroblasts are involved in the closure of three-dimensional wounds, which results in the repopulation of the wound with the cell-derived extracellular matrix [47]. Therefore, we went further with our research study and tested whether hydrogel formulations with incorporated PFs have a cytotoxic effect on the proliferation of human fibroblasts and endothelial cells. The cell viability was determined for cells grown on all hydrogels formulation and compared to the control group—cells grown in conventional tissue culture plate (CTRL) (Figure 7).

The biocompatibility tests for the two cell types (fibroblasts and endothelial cells, respectively) showed that CD hydrogels are non-toxic, with cell viability being over 80% (% of CTRL TCP) in all cases, which means that CD hydrogels do not cause cell death. No significant difference regarding the influence of the hydrogels’ composition on cell proliferation was observed. However, a slight increase in cell viability of endothelial cells can be observed with an increase of the dextran content in the hydrogel matrix, sustained by the biological function of dextran to help cell adhesion and growth [5]. In addition, one can add the contribution of the high interconnectivity of pores, as well as their size and uniformity.

Since in the case of fibroblast cells, the least influence of the hydrogels’ composition on cell viability was observed (Figure 7B), it was decided to perform the following tests only with this type of cells.

#### 2.5.2. Anti-Inflammatory Effect of PFs on Human Cells Exposed to LPS

**Figure 8:**
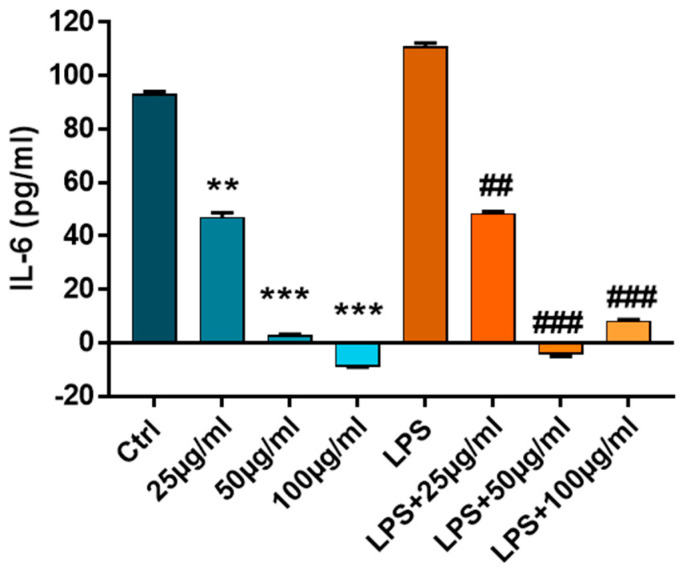
The protein expression of IL-6 released in the conditioned medium from control cells or cells activated with LPS (100 ng/mL) and treated with PFs (25, 50 and 100 µg/mL). *n* = 3, ** *p* < 0.01, *** *p* < 0.001, PFs versus control cells and ## *p* < 0.01, ### *p* < 0.001 PFs versus LPS.

Before testing the anti-inflammatory capacity of PFs-loaded hydrogels, we investigated the effect of three different concentrations of PFs (25, 50 and 100 µg/mL, respectively) on inflammatory cytokine IL-6 released by cells grown in conventional culture, in the presence or absence of LPS (100 ng/mL). For this, human fibroblasts were grown in conventional tissue culture plates and next activated for 18h with LPS in the presence or absence of PFs. The anti-inflammatory effect of PFs was investigated by quantification of IL-6 released by cells in the culture media using ELISA assay (Figure 8).

The results show that PFs exhibit potent anti-inflammatory properties on both control cells and cells exposed to LPS, which are evident even at low concentrations of PF solutions (25 µg/mL), where the reduction of cell inflammation is almost 50% (related to control or LPS). After doubling the concentration of the PFs solution from 25 µg/mL to 50 µg/mL, the reduction of inflammation is almost 100%, so the increase of PFs concentration above this value is not justified.

The concentration of 50 µg/mL of PFs was considered optimal for the anti-inflammatory protective character of PFs in the LPS-induced inflammation process and was further used for encapsulation in hydrogels. Considering this, for the next stage, taking also into account the swelling degree of each type of hydrogel, the CD matrices were embedded with a solution of PFs, so that at the end, they contain approximately the optimal amount of bioactive compound, 50 µg/mL.

#### 2.5.3. Cytocompatibility and Anti-Inflammatory Tests for PFs-Loaded Hydrogel

**Figure 9:**
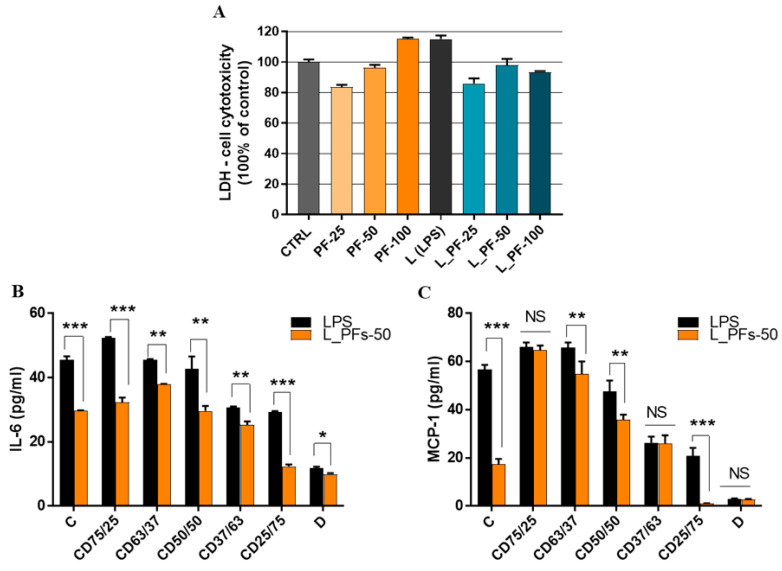
PFs-loaded hydrogels are biocompatible (**A**) and significantly reduce secretion of pro-inflammatory cytokines IL-6 and MCP-1 in human fibroblasts: (**A**) Quantitative evaluation of the cytotoxicity released by fibroblasts cultured on functionalized hydrogels, after 72 h of culture, using LDH assay; (**B**,**C**) IL-6 and MCP-1 concentration released in condition media from cells grown on PFs-hydrogels tested with ELISA assay. Data are presented as a mean ± SD (*n* = 3). An unpaired *t*-test was performed with GraphPad Prism. * *p* ≤ 0.05, ** *p* ≤ 0.01, *** *p* ≤ 0.001, NS–nonsignificant.

The PFs were embedded in different CD hydrogels and before testing the anti-inflammatory effects, the biocompatibility of PFs-functionalized hydrogels was quantified. For this, the fibroblasts were cultured on PFs-embedded hydrogels in the presence or absence of LPS for 72h and cell cytotoxicity was quantified by LDH (Figure 9A).

The biocompatibility results that quantified the level of LDH enzyme released in the cell culture media revealed that after 3 days of culture, PFs-loaded hydrogels exhibited similar cytotoxicity compared to the control (Figure 9A), or even lower, for hydrogels loaded with 25 µg/mL PFs. No statistically significant difference was found between the LDH levels released by the tested hydrogels, indicating that the encapsulation of PFs in hydrogels does not exert an important cytotoxic effect on the cellular component.

To further evaluate anti-inflammatory effects of PFs embedded in hydrogels, the cells were cultured on hydrogels with or without PFs (50 µg/mL), in presence of LPS—to induce an inflammatory status of cells. The ELISA results (Figure 9B,C) showed that the levels of both investigated inflammatory molecules released by cells were reduced when the cells were grown on PFs-functionalized hydrogels. Therefore, the interleukin IL-6 was significantly reduced by all hydrogel formulations with encapsulated PFs (Figure 9B) and the chemokine MCP-1 was significantly reduced by C, CD63/37, CD50/50 and CD25/75 formulations (Figure 9C). All these data reveal that PFs embedded into the hydrogel exhibit anti-inflammatory effects on human cells.

Moreover, from the obtained data it was possible to highlight the anti-inflammatory character of dextran from the hydrogel formulations that do not contain PF, by decreasing the level of the two inflammatory molecules, IL-6 and MCP-1 (Figure 9B,C—black bars). The ability of dextran to reduce the inflammatory response of cytokines is also supported by previous literature [48,49,50]. In our case, by increasing the amount of dextran in the hydrogel formulations, its anti-inflammatory effect overlaps the anti-inflammatory effect of PF. Thus, for the cellulose hydrogel (C), a decrease in the IL-6 level of approximately 35% was observed, due to the effect of the encapsulated PFs, while for the dextran hydrogel (D) this decrease is only 8%, confirming the major anti-inflammatory influence of dextran.

## 3. Materials and Methods

### 3.1. Materials

Microcrystalline cellulose (C) was purchased from Sigma-Aldrich (Saint Louis, MO, USA) under the trade name of Avicel PH-101 (~50 μm particle size; DP = 180). Dextran (D) was obtained by biotechnological methods, using a lactic acid bacteria (LAB) strain identified by 16S rDNA sequence as *Weissella confusa*, isolated in the laboratories of Centre of Advanced Research in Bionanoconjugates and Biopolymers (IntelCentru) of the “Petru Poni” Institute of Macromolecular Chemistry, Iasi [12,40,42]. Polyphenols (PFs) were obtained by extraction from grape seeds of Chambourcin type, according to the procedure described in detail by Ciolacu and coworkers [33]. The identification of the major phenolic compounds from grape seeds led to the following composition: 34.3% gallic acid, 30.8% quercetin, 18.3% monomers anthocyanin, and 9.3% proanthocyanidins. The chemical structures of the phenolic compounds identified in grape seeds are presented in Figure 1. Epichlorohydrin (EPC) was purchased from Merck (Darmstadt, Germany) (purity > 99 %; d = 1.18 g/cm^3^) and was used without further purification. Sodium hydroxide (NaOH) in pellets, with a purity ≥ 97%, was supplied by Merck (Hohenbrunn, Germany).

Cytotoxicity of the hydrogels was tested using a LDH detection kit (ThermoFischer, Rockford, IL, USA), while viability and proliferation of human cells seeded on the hydrogels were quantified using a XTT assay (ThermoFischer, Rockford, IL, USA). Anti-inflammatory effects of hydrogels enriched with polyphenolic compounds on human cells stimulated with lipopolysaccharide (LPS, Sigma-Aldrich) were tested by quantification of inflammatory molecules MCP-I and IL-6 using Enzyme-Linked Immunosorbent Assays (DuoSet ELISA Kits, R&D Systems, Minneapolis, MN, USA).

### 3.2. Methods

#### 3.2.1. Preparation of the Hydrogels

3D matrices were prepared from cellulose (C) and from dextran (D), but also from the mixture of these two polysaccharides, in different gravimetric ratios (CD), C/D: 75/25, 63/37, 50/50, 37/63 and 25/75 (Table 1). The hydrogels were prepared as follows: 0.25 g polymer was added to 3.67 mL 8.5% NaOH solution and frozen at low temperature (−30 °C). After thawing, 1.45 mL EPC was added under continuous stirring. The obtained composition was maintained for 5 h at 84 °C. The hydrogels were washed with warm distilled water (60 °C), for 15 days, in order to remove the excess of NaOH, any EPC traces and NaCl, followed by drying by lyophilization.

#### 3.2.2. Swelling Measurements

Swelling studies were performed for all hydrogels in distilled water, at 37 °C. The samples were periodically removed from the swelling medium, gently wiped with a soft tissue to remove the surface solution, quickly weighed and then placed back into the vessel. The swelling degree (Q_max_, %) of the hydrogels was determined gravimetrically and calculated according to Equation (1):

(1) $$Q_{\max}=\frac{M_{s}-{\;M}_{d}}{M_{d}}\;\cdot 100\%$$

where: M_s_—the weight, at time t, of swollen hydrogel (g); M_d_—the weight of dry hydrogel (g).

The equilibrium swelling degree (Q_eq_, %) was determined for the never-dried hydrogels (hydrogels just after synthesis) using Equation (2):

(2) $$Q_{\mathrm{eq}}=\frac{M_{\infty }-{\;M}_{d}}{M_{d}}\;\cdot 100\%$$

where: M_∞_—the weight of swollen hydrogel, at equilibrium (g); M_d_—the weight of dry hydrogel (g).

In order to determine the kinetics of solvent diffusion into the hydrogels matrices, Equation (3) was used to describe the Fickian or non-Fickian behavior of swelling-controlled release systems [33]:

(3) $$\frac{W_{t}}{W_{\mathrm{eq}}}=k_{sw}\cdot t^{n_{sw}}$$

where: W_t_—the amount, at time t, of water absorbed by the hydrogel (g); W_eq_—the amount of water absorbed by the hydrogel at equilibrium (g); *k_sw_*—the swelling constant that incorporates the characteristics of the macromolecular network system (min^−1^); *n_sw_*—the swelling diffusional exponent, which is indicative of the transport mechanism. The constants *n* and *k* were calculated from the slopes and intercepts of the plots of ln(W_t_/W_eq_) vs. ln(t). Equation (3) was applied in the early swelling stages (swelling degree less than 60%) where the linearity was observed.

#### 3.2.3. Incorporation of the PFs

**Figure 10:**
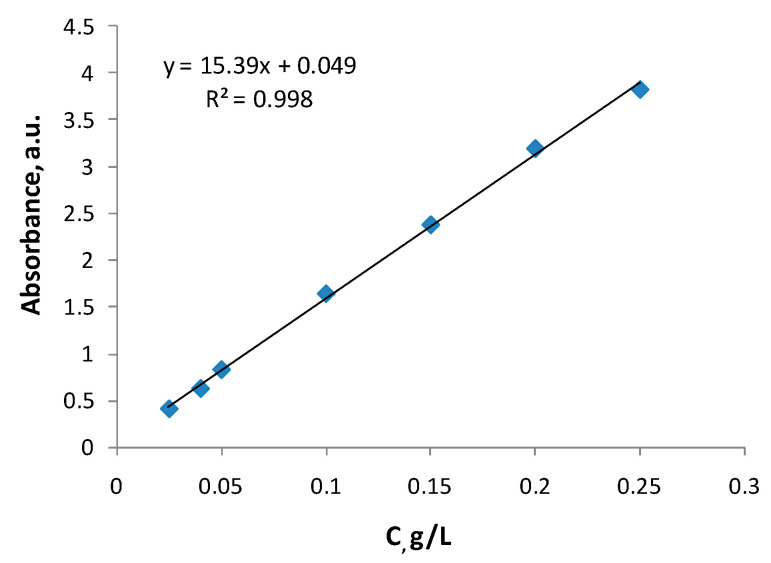
Calibration curve of PFs (water:ethanol = 19:1, 37 °C).

0.1 g dried hydrogel was immersed in 25 mL solution of 4 g/L PFs in water:ethanol (19:1, *v*/*v*) and left to swell at room temperature for 72 h, while the PFs penetrated and/or attached to matrices. Finally, the PFs-loaded samples were dried by lyophilization. In order to establish the amount of PFs incorporated in the hydrogels, the remaining solutions were analyzed by UV-VIS spectroscopy, measuring the absorbance at 280 nm. The calibration curve (Figure 10) was obtained according to different concentrations of PFs in water-ethanol (19:1, *v*/*v*), in the range of 0.025 ÷ 0.25 g/L. The unknown concentrations of the remaining solutions after PFs incorporation were determined using the equation obtained from the calibration curve, y = 15.39x + 0.049, with a correlation coefficient of R^2^ = 0.998.

The incorporation degree (I_d_) of PFs into hydrogel matrices was calculated using Equation (4):

(4) $$I_{d}=\frac{c_{0}V_{0}-\mathrm{cV}}{M}\;\cdot 100\%$$

where: c_0_—the initial concentration of the PFs solution (g/mL); V_0_—the volume of initial PFs solution (mL); c—the concentration of remained PFs in solution, determined from the calibration curve (g/mL); V—the volume of the PFs solution remaining after incorporation (mL); M—the weight of dry PFs loaded-hydrogel (g).

#### 3.2.4. In Vitro Release of the PFs

In vitro release studies have been conducted by a standard dissolution procedure using the water:ethanol (19:1, *v*/*v*) mixture as release medium, at 37 °C [33]. 1 mL samples of release medium were withdrawn periodically, at predetermined time intervals, and the absorbance at 280 nm was measured. In order to maintain the solution concentration, the sample was reintroduced in the system after analyzing. The PFs concentration in release medium was calculated based on the PFs calibration curve (see Figure 10).

In order to kinetically analyze the release data of PFs from hydrogel matrices with different compositions, a semi-empirical equation (Equation (5) of Korsmeyer–Peppas release model was used and applied at the earlier stages of PFs release (between 5% and 60% fractional release) [35]:

(5) $$\frac{M_{t}}{M_{\infty }}=k\cdot t^{n}\;$$

where: M_t_—the amount of PFs released at time t (g); M_∞_—the amount of PFs at the equilibrium state (g); *k*—the rate constant, characteristic to the PFs-polymers system, incorporating its structural and geometric character (min^−1^); *n*—the diffusional exponent, which suggests the nature of the release mechanism. The value of *n* depends on the hydrogel shape. Thus, for a cylinder shape, if: (i) *n* < 0.45 then a Fickian diffusion release mechanism is implied, (ii) 0.45 < *n* < 0.89, the release mechanism follows an anomalous transport mechanism and (iii) *n* is above 0.89 then Case II release mechanism takes place [32,35].

#### 3.2.5. Cell Isolation and Culture

*Endothelial cells*: human valvular endothelial cells isolated from non-calcified cusps of human aortic valves as we previously described [51]. Endothelial cells were cultured in endothelial cell growth medium with 20% FBS (Gibco) and 100 U/mL Penicillin, 100 μg/mL Streptomycin and 50 μg/mL Neomycin (Sigma-Aldrich, Schnelldorf, Germany).

*Fibroblasts*: human valvular interstitial cells—fibroblast-like cells—purchased from Innoprot (no. P10462), and cultured in Dulbecco’s Modified Eagle Medium (DMEM, Gibco), supplemented with 10% Fetal Bovine Serum (FBS, Gibco) and 1% Antibiotics (Penicillin/Streptomycin, Sigma-Aldrich, Germany), according to manufacturer’s protocols.

#### 3.2.6. XTT—Cell Viability Assay

The dried hydrogels composed of different amounts of cellulose and dextran, with or without PFs were sectioned and UV sterilized for 30 min on both sides. Once sterilized, they were hydrated with complete medium (DMEM or M200, 10% FBS and 1% P/S) in a 96-well plate for 3 h, at 37 °C, 5% CO_2_. After hydrogel hydration, human fibroblasts and endothelial cells were seeded at a density of 7500 cells/hydrogel in a 96-well plate. After 72 h, the biocompatibility of the hydrogels was determined by XTT assay and the viability of cells from the hydrogels was compared to the viability of cells that were grown in normal culture conditions, in a conventional tissue culture plastic 96-well plate (control group—Ctrl TCP). Live cells reduce the yellow tetrazolium salt to water-soluble orange colored formazan, which is spectrophotometrically measured at 450 nm.

#### 3.2.7. LDH—Cytotoxicity Assay

To test the cytotoxicity of PFs extracted from grape seeds, cells were cultured at a density of 7500 cells/well in a 96-well plate and exposed to different concentrations of PFs (25–100 µg/mL), in the presence or absence of LPS (100 ng/mL). After 24 h, the medium was collected and the lactate dehydrogenase enzyme (LDH) activity—an enzyme that is released into the culture medium when the cell membrane is damaged—was quantified according to the manufacturer’s protocol.

#### 3.2.8. ELISA Assay

To test the anti-inflammatory properties of PFs extracted from grape seeds, the human cells were cultured in conventional culture or on the hydrogels and exposed to different concentrations of PFs (25–100 µg/mL), in the presence or absence of LPS (100 ng/mL). After 24 h, the medium was collected and the levels of inflammatory cytokine MCP-1 and IL-6 released by cells were quantified using ELISA DuoSet kits (R&D systems, Abingdon, UK), following manufacturer’s instructions.

### 3.3. Equipment

UV–VIS absorption spectra were recorded at room temperature on a Hewlett Packard 8540A UV–VIS spectrophotometer, in 10 mm quartz cells, using water:ethanol mixture as a solvent.

Attenuated Total Reflection Fourier Transformed Infrared (ATR-FTIR) spectroscopy was carried out on silicon single-crystal parallelepiped internal reflection elements (IRE; 55 × 5 × 2 mm, 45° incident angle), using a Bruker Vertex 70 instrument. All the spectra were the results of 256 scans at a resolution of 4 cm^−1^, frequency range 4000–400 cm^−1^.

The morphology of the samples was investigated by using a Scanning electron microscope (SEM) type Quanta 200, operating at 30 kV with secondary electrons, in low vacuum mode.

The average pore size and the standard deviation (SD) were determined using the SEM images exported in the image analysis software—ImageJ, measuring 20 randomly chosen pores and following the steps presented in the procedure described in the literature [52,53].

The cytotoxicity and cytocompatibility experiments of hydrogels, PFS and PFs loaded-hydrogels were carried out by Tecan Infinite M200 Pro spectrophotometer measurements.

## 4. Conclusions

New 3D bicomponent matrices based on cellulose and dextran, for the encapsulation and controlled release of anti-inflammatory bioactive compounds have been obtained. The properties of the hydrogels were found to be dependent on their composition. Thus, the presence of dextran in different proportions has a positive impact on the hydrogel’s morphology, by increasing the uniformity and interconnectivity of the pores. In addition, the hydrogels CD50/50, CD37/63 and CD25/75, respectively, show a high swelling degree and an encapsulation capacity of PFs clearly superior to cellulose hydrogel. The PFs release kinetics indicate different transport mechanisms depending on hydrogels’ composition and morphology. For instance, the PFs release from D hydrogel follows a Fickian diffusion mechanism, while the diffusional coefficient value obtained for C hydrogel indicates a non-Fickian mechanism Case II based on the swelling or relaxation of polymeric chains. Related to CD hydrogels, these exhibit a non-Fickian model with anomalous transport mechanism governed by diffusion and swelling processes with comparable rates.

The cell-based experiments showed that fibroblasts and endothelial cells were successfully cultured on the CD hydrogels with a high viability, of over 80% for all formulations, a fact which demonstrates their good cytocompatibility. Moreover, the encapsulation of PFs as a bioactive principle gives to hydrogels anti-inflammatory properties, sustained by the anti-inflammatory tests in the presence of LPS, used as a pro-inflammatory model.

Considering the results obtained, it can be concluded that PFs-loaded CD hydrogels have the potential to facilitate and accelerate wound healing by inhibiting the inflammation process through a controlled and sustained release of the anti-inflammatory bioactive compounds in skin wound applications.

## Figures and Tables

**Table 1:** The composition of cellulose-dextran hydrogels and their main features.

Samples	Hydrogels Composition		Hydrogels Features	
	Cellulose, %	Dextran, %	Gel Fraction Yield, %	Q_eq_, %
C	100	0	99.9	1640
CD75/25	75	25	99.9	2980
CD63/37	63	37	95.2	3530
CD50/50	50	50	90.4	4430
CD37/63	37	63	87.8	4460
CD25/75	25	75	84.9	4520
D	0	100	76.3	5310

**Table 2:** Swelling kinetic parameters for different hydrogel formulations.

Samples	Swelling Kinetic Parameters			Q_max_, %
	n_sw_	k_sw_	R^2^	
C	0.192	0.712	0.993	1064
CD 75/25	0.079	0.544	0.995	1485
CD 63/37	0.087	0.436	0.997	1363
CD 50/50	0.183	0.869	0.999	1607
CD 37/63	0.205	1.226	0.997	1873
CD 25/75	0.269	1.417	0.992	2367
D	0.369	2.207	0.993	3745

**Table 3:** The average pore size of the obtained hydrogels.

Samples	Average Pore Size, μm
C	66.1 ± 12.0
CD 75/25	44.4 ± 9.2
CD 63/37	44.0 ± 9.8
CD 50/50	41.6 ± 8.6
CD 37/63	34.8 ± 7.8
CD 25/75	27.1 ± 7.0
D	13.5 ± 3.1

**Table 4:** ATR-FTIR spectral characteristics for cellulose (C), dextran (D) and CD hydrogels.

Samples	HBI	A_1630_/A_2900_	A_1116_/A_2900_	A_1066_/A_2900_	A_1116_/A_761_	A_1066_/A_761_
C	2.426	0.695	2.253	2.536	19.86	22.36
CD 75/25	2.537	0.588	2.250	2.544	13.27	15.00
CD 63/37	2.267	0.694	2.119	2.362	10.43	11.63
CD 50/50	2.515	0.631	2.138	2.404	9.45	10.62
CD 37/63	2.216	0.675	1.906	2.098	8.00	8.80
CD 25/75	1.976	1.169	1.495	1.557	5.71	5.95
D	1.694	1.379	1.358	1.379	4.27	4.34

**Table 5:** ATR-FTIR spectral characteristic for CD hydrogels, with or without PFs.

PFs-Loaded Hydrogels	A_1522_/A_2900_	A_1705_/A_2900_	I_d_, %
C + PFs	0.591	0.337	13.6
CD75/25 + PFs	0.612	0.366	22.1
CD50/50 + PFs	0.637	0.418	24.5
CD25/75 + PFs	0.705	0.459	33.7
D + PFs	0.710	0.469	41.9
PFs	0.789	0.544	-

**Table 6:** Kinetic parameters of PFs release for C, D and CD hydrogels.

PFs Loaded-Hydrogels	*n*	*k*	R^2^	Transport Mechanism
C	1.004	1.009	0.984	Case II
CD 75/25	0.573	1.205	0.993	Anomalous
CD 63/37	0.563	1.369	0.990	Anomalous
CD 50/50	0.558	1.369	0.993	Anomalous
CD 37/63	0.537	1.535	0.992	Anomalous
CD 25/75	0.504	1.761	0.993	Anomalous
D	0.329	2.630	0.994	Pseudo Fickian diffusion

## Data Availability

Data are contained within the article.
